# Effects of an Advanced Practice Nurse In-Home Health Consultation Program for Community-Dwelling Persons Aged 80 and Older

**DOI:** 10.1111/jgs.12026

**Published:** 2012-11-29

**Authors:** Lorenz Imhof, Rahel Naef, Margaret I Wallhagen, Jürg Schwarz, Romy Mahrer-Imhof

**Affiliations:** * School of Health Professions, Institute of Nursing, Zurich University of Applied SciencesWinterthur, Switzerland; † School of Nursing, University of California at San FranciscoSan Francisco, California; ‡ Department of Business, Lucerne University of Applied Sciences and ArtsLucerne, Switzerland

**Keywords:** advanced practice nursing, health consultation, home visit, oldest old, community nursing, fall prevention, hospitalization

## Abstract

**Objectives:**

To evaluate the effects of an advanced practice nurse (APN) in-home health consultation program (HCP) on quality of life, health indicators (falls, acute events), and healthcare utilization.

**Design:**

Randomized clinical trial.

**Setting:**

One urban area in the German-speaking part of Switzerland.

**Participants:**

Four hundred sixty-one community-dwelling individuals aged 80 and older (mean age 85, 72.7% female, all Caucasian) participated in the intervention (n = 231) and control (n = 230) groups.

**Intervention:**

After a comprehensive geriatric assessment, participants were randomly assigned to the 9-month HCP with four in-home visits and three phone calls from APNs or to a control group with standard care with no intervention.

**Measurements:**

The primary outcome was quality of life at 3, 6, and 9 months. Secondary outcomes were incidence of falls, acute events due to health problems, and healthcare utilization measured for 3-month periods at 3, 6, and 9 months.

**Results:**

The intervention and control groups did not differ significantly on any dimension of the World Health Organization Quality of Life questionnaire but differed significantly over 9 months in self-reported acute events (116 vs 168, relative risk (RR) = 0.70, *P* = .001), falls (74 vs 101, RR = 0.71, *P* = .003), consequences of falls (63.1% vs 78.7%, chi-square = 7.39, *P* = .007), and hospitalizations (47 vs 68, RR = .70, *P* = .03).

**Conclusion:**

The in-home HCP provided by APNs and guided by the principles of health promotion, empowerment, partnership, and family-centeredness, can be effective in reducing adverse health outcomes such as falls, acute events, and hospitalizations.

The majority of individuals aged 80 and older live in communities, where they are responsible for managing their chronic conditions and any limitations that may complicate their daily lives. To avoid acute episodes or hospitalization, these older individuals require the knowledge and skills necessary to recognize symptoms of destabilization, negotiate support from social networks, create a safe environment, and decide when to use services that the healthcare system provides.^1^,^2^ These skills play a crucial role in maintaining health, quality of life, and independent living in the community.

Programs aimed at promoting self-care and disease-management skills in persons with particular conditions, such as diabetes mellitus or cardiovascular diseases, have shown promising results in community and primary care settings.^3^–^8^ In addition, programs that focus on specific symptoms or problems such as individual falls are also effective in community-dwelling older persons,^9^,^10^ but programs that focus on a general population of older people living at home have produced controversial findings about the efficiency of these preventive home visits.^11^–^14^ Because frail older persons and persons with disabling health conditions are often excluded from preventive health program studies,^15^ the effects of such health conditions on functional impairment or healthcare utilization remain unclear in this heterogeneous population. It has been suggested that further research is needed that focuses not only on client groups that benefit most, but also on elderly populations in general, using outcomes of quality of life, mental health, social support, and caregiver burden.^15^,^16^

Many chronic illness management programs use advanced practice nurses (APNs) who, given their superior knowledge and skills, are well positioned to deliver and coordinate care that aims to improve the self-management abilities and health competencies of older persons and thus enhance their quality of life.^7^,^8^,^17^–^22^ With the international rise in the roles assumed by APNs,^23^,^24^ research is needed that tests the effectiveness of APN-led community-based programs that are complementary to, and done in collaboration with, the interprofessional healthcare team. The current study examined a new in-home health consultation program (HCP) implemented by APNs for a population of individuals age 80 and older living at home. The HCP comprises a standardized comprehensive geriatric assessment, evidence-based guidelines for prevalent health problems in elderly adults, four in-home consultations customized to individual needs, and three follow-up telephone calls over 9 months. To the knowledge of the authors, this is the first study investigating the use of APN health consultation to promote self-care ability and skills for a home-based population aged 80 and older. The main aim of this study was to evaluate the effectiveness of the HCP in terms quality of life, health indicators, and healthcare utilization. It was hypothesized that the HCP would increase quality of life, reduce adverse health outcomes (falls, acute events), and reduce healthcare utilization (hospitalization).

## Methods

### Study Design

A prospective, randomized clinical trial (2008–2011) was undertaken to determine the effect of the 9-month in-home HCP implemented by APNs on quality of life, selected health indicators, and healthcare utilization in community-dwelling persons aged 80 and older. This article reports the main findings; an in-depth description of HCP itself is reported elsewhere.^25^

### Settings and Participants

The study was conducted in one of the major cities in the German-speaking part of Switzerland. It involved a convenience sample of German-speaking, community-dwelling persons aged 80 and older who were cognitively able to understand and consent to the study. Persons at the end of life or with a major psychiatric diagnosis or severe cognitive impairment, as measured using the Clinical Dementia Rating Scale, were excluded.^26^ The ethics commission of the Canton of Zurich approved the study (Ref 06/08.08.2008).

**Figure 1:**
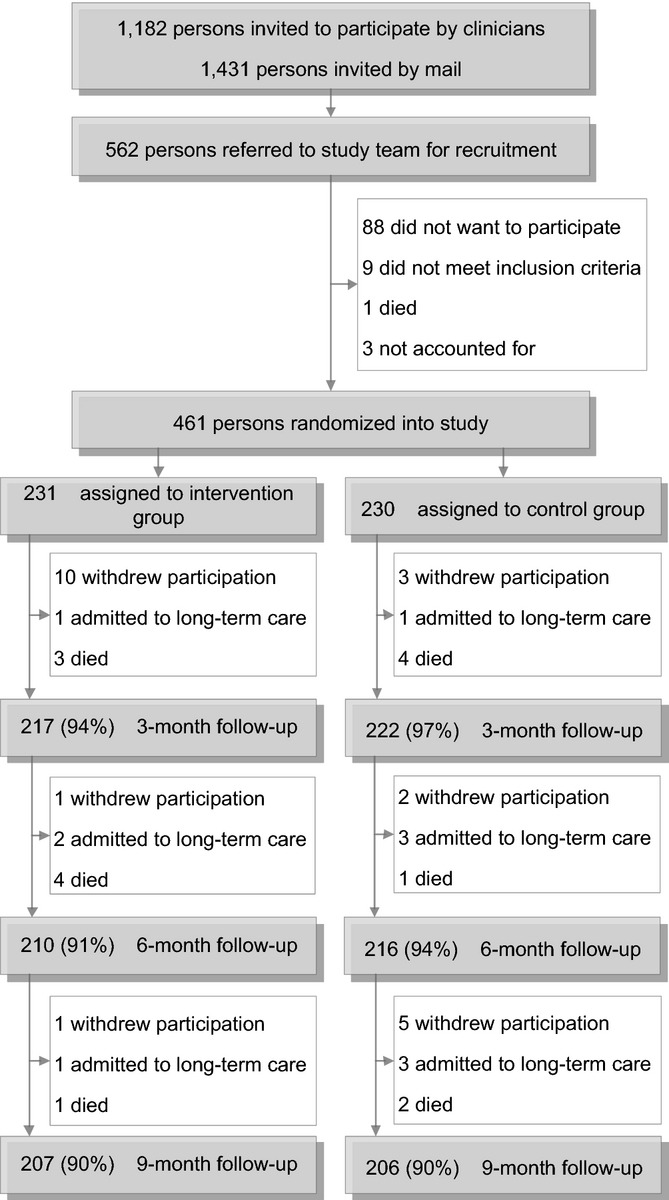
Participant flow.

Various health organizations such as local hospitals, home care organizations, and church social services and by community nurses and family physicians extended the invitation to participate in the study to 1,182 potential participants. An additional 1,431 letters of invitation were sent to people's homes. Fifty-five percent (n = 2,613) of the city's inhabitants aged 80 and older and living at home were invited to take part in the study. The response rate was 21.5%, with 562 persons expressing an interest in the study. The study team informed those who expressed an interest about the study procedures (Figure 1). The convenience sample included 10% of the city's population aged 80 and older and living at home. The study sample had a higher percentage of women (72.7%) than the city's overall female population (67.7%). Age distribution in the study was not statistically significantly different from that of the city's inhabitants aged 80 and older.^27^ Female participants were less likely to be in a partnered relationship than the whole Swiss population of the same age (aged 80–84, 29% vs 32%; aged 85–89, 14% vs 18%; aged ≥90+, 4% vs 10%).^28^ Therefore, the sample was fairly representative of elders in the region.

### Randomization

Two assessment visits by the APNs were used to collect baseline data before randomization. With the permission of participants, collected health information was forwarded to their family physicians and, if they received home care nursing, also to the home care nurse. After the second assessment visit, participants were randomly assigned to the intervention or the control group using a computer-generated list of random numbers with a one-to-one sequence. A person who was not involved in the recruitment of study participants or data collection prepared sealed envelopes with group assignment. The APN opened the envelope at the end of the visit, and the participant was informed about group allocation.

### Intervention

**Figure 2:**
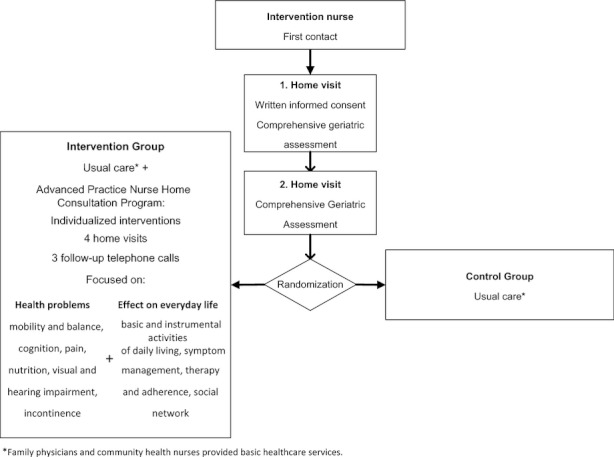
Intervention.

Persons in the control and intervention groups received healthcare services as usual provided by community health nurses (23%) and physicians (97%) and covered by the participants' mandatory health insurance (Figure 2). Persons randomized to the intervention group took part in a complementary 9-month in-home HCP delivered by one of four APNs.^25^ The APNs were all registered nurses with a master's degree in Nursing Science. The nurses were prepared for a generalist practice with a role that was similar to that of a clinical nurse specialist. The four nurses had an average of 22 years of work experience in home care and gerontological nursing. A collaborating doctor specialized in geriatrics trained them for the intervention program in comprehensive geriatric assessment. To ensure continuity, the same APN who conducted the prerandomization assessment delivered the intervention. Three measures were taken to establish consistency among the four intervention nurses. First, APNs were trained for the intervention in a 5-day training program. The consultation followed a standardized sequence of decisions that considered the health problems that the nurse identified and the concerns of the participant. Second, the project team obtained and carefully reviewed a detailed intervention protocol. Discrepancies in documentation or standardization were discussed among the intervention nurses, and decisions for further procedures were made. Third, the intervention nurses participated in regular clinical briefing sessions.

The intervention included four home visits (mean length 46 ± 6 minutes) after 4, 12, 24, and 36 weeks, and three telephone calls (mean length 17 ± 4 minutes) after 8, 18, and 30 weeks. Total intervention time per participant averaged 4 hours.

The HCP was developed based on the principles of health promotion, empowerment, partnership, and family-centeredness, as described in behavioral change theories.^25^,^26^,^29^,^30^ The interventions were customized to the participants' needs. The intervention nurses used evidence-based guidelines regarding prevalent health concerns such as mobility, vision and hearing, pain, nutrition, cognitive abilities, and bladder control, along with questions of social support and case management, to address the health problems they had identified and the concerns on which participants had chosen to focus.^14^,^31^–^37^

In 87% of the interventions, participants focused on their family situation and on maintaining or improving their relationship with their next of kin. Problems with mobility (85%), pain management (70%), healthy nutrition (48%), and hearing and vision (45%) were the other major health-related concerns that were addressed. In 44% of the interventions, how to best make use of the healthcare system, including necessary home visits by the physician or organizing adjuvants, was discussed.

At the end of each visit, participants developed an action plan with concrete activities or strategies to address their health or family concerns. This action plan was evaluated during the following visit or telephone call and served as a basis for further interventions. During the intervention time with participants, nurses engaged in assessment of health and family situation; education and counseling regarding specific health concerns, daily management of symptom or illness, and organization of family or professional care; performing activities that the participant was unable to perform alone; skills training; and evaluation of previous nurse activities and activities that participants had decided to do.

### Data Collection and Measurements

The APN collected data pertaining to the way the intervention was administered, the consultation procedures, and the participants' goals using standardized intervention protocols. Outcome measures were obtained using questionnaires and structured telephone interviews after 3, 6, and 9 months. Participants were contacted by telephone if the questionnaires were not returned in time or were incomplete. Research assistants who were not involved in the delivery of the intervention managed data collection for outcome measures. Initially, they were blinded for their data collection, but they subsequently learned about group allocation as the participants described their experiences during the telephone interviews.

#### Baseline Measures

The written questionnaire included 76 items from validated instruments assessing quality of life (World Health Organization Quality of Life Assessment short version, WHOQOL-Bref^38^), independence in daily activities (Older Americans Resources and Services, OARS^39^,^40^), and items that assessed social support, self-efficacy, and family functioning. The latter variables are not reported in this article. All questionnaires were returned in the mail before randomization. The multidimensional geriatric nursing assessment^25^ covered demographic variables, living situation, family network, and health status (mobility and falls, pain, vision and hearing ability, sleep pattern, bladder control, nutritional status, substance use, cognition, and use of medications and aides for mobility). For these purposes, clinical tests were included for vision (Amsler-Gitter Test), gait, balance, and strength (Timed Up and Go Test,^41^) tandem stand, timed five-chair-rise test,^42^ and screening for malnutrition (Mini Nutritional Assessment^43^), and depression (Geriatric Depression Scale GDS-4^44^).

#### Primary Outcome Measures

The primary outcome was quality of life, measured using the German version of the WHOQOL-Bref,^38^,^45^ which includes 26 items that are rated on five 5-point Likert scales(very poor to very good, very dissatisfied to very satisfied, not at all to an extreme amount, not at all to extremely, never to always). The WHOQOL-Bref yields a score for general quality of life in each of four domains—physical, psychological, social, and environmental—with a score of 100 indicating maximum quality of life. Internal consistency for the subscales ranges between an alpha of 0.70 and 0.86.^45^

#### Secondary Outcome Measures

Secondary outcomes included incidence of acute events, falls, hospitalization, and healthcare use. These outcomes were obtained in telephone interviews. All variables were binary (yes vs no).

Acute events were defined as acute health symptoms that required action. Participants were asked, “Did you experience acute health difficulties within the last 3 months?” For every participant, three 3-month periods were observed consecutively (month 0–3, month 3–6, and month 6–9). To describe the type of acute events, health problems were categorized into 13 predefined categories: cardiovascular, orthopedic, gastrointestinal, pulmonary, rheumatic, nephrological, neurological, urological, ophthalmological, dermatological, endocrinological, oncological, and other problems.

Falls were assessed with the question: “Did you fall within the last 3 months?”

Hospitalization was defined as a planned or unplanned hospital stay or an admission to the emergency department of a hospital. Participants were asked: “Have you been in a hospital or an emergency department within the last 3 months?”

The use of various professional services or consultations such as home care, pharmacists, and family physicians was assessed by asking participants: “Did you receive or consult with [particular service or health professional] within the last 3 months?” for each type of service.

### Statistical Analysis

Baseline data are reported as means±standard deviation (continuous data) or percentages (categorical data), depending on the data level. The analysis was based on intention to treat. There was a low rate of dropouts and missing data (10.4%, n = 48).

Multilevel analyses, which take into account the hierarchical data structure of a randomized clinical trial design and allow for the analysis of changes in the outcome variables over time on an individual level, were performed to determine the effect of the intervention on the outcome.^46^

There were two levels of differences. On level one (within person) the individual change trajectory was modeled to describe how each participant's status depended upon time. On level two (between person) interindividual differences in change were modeled to describe how features of the change trajectories varied between participants. Intraclass correlation (ICC) was calculated as the share of total variation that the intervention explained.^47^

Linear mixed modeling was used to analyze continuous variables with an approximately normal distribution such as quality of life. Generalized linear mixed modeling was used for variables that were not normally distributed or with a binary answer scale.

Data were analyzed using SPSS version 19 (SPSS, Inc., Chicago, IL). Two-sided tests with the significance level set at <.05 were used to test the hypotheses. In addition, the occurrence of falls, acute events, and hospitalizations in each time measurement interval was compared using the chi-square test.

## Results

### Participants

Four hundred sixty-one persons (82% of the people who expressed an interest in participating) agreed to participate. Two hundred thirty-one were subsequently randomized to the intervention group and 230 to the control group. Of the 461 persons who began the study, 413 (90%) completed the 9-month follow-up (Figure 1). Within the 9-month study period, 22 persons withdrew, 11 entered a long-term care facility, and 15 died. There was no statistically significant difference between the intervention and control groups in number of drop-outs or reasons for dropping out.

### Baseline Characteristics

The characteristics of the two study groups were comparable at baseline (Table 1). The mean age of the participants was 85, 73% were female, 67% lived alone, 63% were college graduates or had a professional degree, and 89% were financially comfortable.

**Table 1:** Baseline Characteristics

Characteristic	Intervention (n = 231)	Control (n = 230)	*P*-Value
Age, mean ± SD	85 ± 4	85 ± 4	.88
Female, n (%)	167 (72.3)	168 (73.0)	.86
Living alone, n (%)	159 (68.8)	150 (65.2)	.36
Education <10 years, n (%)	63 (27.3)	65 (28.3)	.70
Financial situation difficult, n (%)	25 (11.0)	26 (11.3)	.61
Activities of daily living (Older Americans Resources and Services), mean ± SD	24.5 ± 3.3	24.4 ± 3.5	.62
Health			
Body mass index, kg/m^2^, mean ± SD	24.8 ± 4.3	24.7 ± 3.8	.74
Mini Nutritional Assessment score ≥12, n (%)	178 (77.1)	177 (77.0)	>.99
Cardiological and pulmonary problems, n (%)	95 (41.1)	99 (43.0)	.68
Daily pain, n (%)	70 (30.3)	70 (30.4)	.93
Sleeping problems, n (%)	102 (44.2)	104 (45.2)	.22
Incontinence, n (%)	61 (26.4)	79 (34.3)	.06
Amsler-Gitter vision test normal, n (%)	136 (65.1)	135 (66.5)	.71
Increase in forgetfulness within previous 3 months, n (%)	66 (28.6)	75 (32.6)	.57
Effect of forgetfulness on activities of daily living, mean ± SD^a^	24.4 ± 18.1	29.3 ± 22.1	.16
Self-rated health good to excellent, n (%)	143 (61.9)	139 (60.5)	.97
Geriatric Depression Scale score <1, n (%)	185 (80.4)	182 (79.1)	.73
World Health Organization Quality of Life, mean ± SD			
Total score	69.6 ± 17.9	68.8 ± 16.8	.60
Physical	68.6 ± 18.0	68.4 ± 16.1	.91
Psychological	73.8 ± 14.3	72.1 ± 14.3	.23
Social	75.7 ± 14.5	72.9 ± 14.7	.04
Environmental	79.7 ± 13.6	79.7 ± 12.7	>.99
Mobility, n (%)			
Falls within the last 12 months	86 (37.2)	100 (43.5)	.17
Walk daily >30 minutes	158 (83.1)	151 (84.8)	.53
Timed Up and Go Test time <10 seconds	68 (30.4)	62 (28.1)	.59
Tandem stand score ≤1	152 (69.4)	146 (67.0)	.63
Timed 5-chair-rise test ≤18.2 seconds	171 (85.5)	164 (83.2)	.54
Use of healthcare services, n (%)			
Home care or district nurse	51 (22.1)	55 (23.9)	.48
Family physician >4 visits/yr	152 (65.8)	155 (67.4)	.27
Hospitalization within last 12 month	79 (34.2)	72 (31.3)	.64
Family support hours/wk, mean ± SD	21.2 ± 47.0	24.1 ± 50.5	.51

SD = standard deviation.

a Analog scale 0 (no effect) to 100 (very strong effect).

Quality of life was high (mean 69.2 ± 17.3), approximately 10.7 points above the German norm rate for this age group.^45^ Almost two-thirds of the participants rated their health as good to excellent, although 30% experience pain regularly and 27% occasionally. Participants reported sleeping problems (45%), experienced cardiological and pulmonary symptoms (42%), and used prescribed medications (95%, mean 4.6 ± 3.1 drugs).

Approximately 34% of participants were able to manage their household independently. As many as 57% needed regular support from informal caregivers or home care services, and 9% were completely dependent on daily support from family members or community nurses. The value for independent daily living (OARS) was measured at baseline and after 9 months. OARS scores did not differ significantly between the intervention (mean 24.5 ± 3.3) and control (mean 24.4 ± 3.5) groups at baseline, and there was no significant change over time in either group.

### Quality of Life

It was hypothesized that the nursing intervention would improve at least some aspects of the participants' quality of life, but the multilevel analysis showed an inconsistent pattern. Overall, it was not possible to infer a clear statement about the differences in general (*P* = .92), physical (*P* = .10), psychological (*P* = .05), social (*P* = .04, which was also significant at baseline), or environmental (*P* = .11) quality of life between the intervention and control groups after the 9-month intervention (Table 2).

**Table 2:** Multilevel Analyses

		Predictor		
		
		Intervention	Time Course	Interaction^a^
		
Outcome	ICC (%)	*P*-Value for Fixed Effects		
Quality of life				
Overall	57.6	.92	.47	.51
Physical	76.3	.10	.04	.17
Psychological	76.8	.05	.01	.13
Social	55.9	.04	.46	.14
Environmental	68.5	.05	.65	.11
Acute events	20.4	.002	.17	.85
Incidence of falls	22.0	.001	.19	.02
Hospitalization	21.4	.86	.33	.24
Pharmacist counseling	36.6	.002	.004	.14

Random effects are not reported but were used to calculate intraclass correlation (ICC) and to assess the multilevel analysis models.

a Interaction term of intervention and time course. Sample size varies depending on the number of missing values.

### Acute Events

The multilevel analysis showed a significant difference in the incidence of acute events between the control and intervention groups during the 9-month period. ICC accounted for 20.4% of the variance, and the intervention was significant in the model (fixed effect, *P* = .002). Time course and interaction term had no significant fixed effect.

During the intervention, the number of 3-month periods with at least one acute event was lower in the intervention group (n = 116) than in the control group (n = 168, RR = 0.70, number needed to treat (NNT) = 4.3). Overall, orthopedic (25%), cardiovascular (11.4%), gastrointestinal (11.2%), rheumatic (6.8%), and neurological (5.9%) problems were mentioned as the most common reasons for acute events in the two groups (Table 3).

**Table 3:** Comparison of Intervention and Control Groups

	Intervention	Control			
				
Outcome	n (%)		Relative Risk	Number Needed to Treat	*P*-Value
Acute events	116 (53)	168 (76)	0.70	4.3	<.001
Falls	74 (34)	107 (48)	0.71	7.1	.003
Hospitalization	47 (23)	68 (33)	0.70	10.0	.03

### Incidence of Falls

In the year preceding the study, 37% of the participants experienced a reduction in their mobility, and 40% fell at least once. At baseline, there was no significant difference between the control and intervention groups in number of falls over the previous year, in the results of the Timed Up and Go Test, in balance (tandem stand), or in the percentage of participants who walked more than 30 minutes a day (Table 1).

The multilevel analysis showed a significant reduction in the number of falls in the intervention group during the 9-month period. ICC accounted for 22.0% of the variance, and the intervention was significant in the model (fixed effect, *P* = .001). The interaction term was also significant, whereas the time course had no significant effect.

During the intervention, the number of 3-month periods with falls was significantly lower in the intervention group (n = 74) than in the control group (n = 107, RR = 0.71, NNT = 7.1, *P* = .003). When the consequences of falls, such as fractures, hematomas, open wounds, or pain for several days, were considered, the intervention group had a significantly lower percentage of falls with consequences (63.1%) than the control group (78.7%, chi-square = 7.39, *P* = .007).

### Healthcare Utilization

At study entry, 33% of participants (n = 151) reported at least one hospitalization within the previous year. The number of hospitalizations did not differ significantly between the intervention (34%) and control (31%) groups in the year preceding the study (Table 1).

The multilevel analysis revealed no significant effects of intervention, time course, or interaction term, although during the intervention, the number of 3-month periods with hospitalization was significantly lower in the intervention group (n = 47, 23%) than in the control group (n = 68, 33%, *P* = .03, RR = 0.70, NNT = 10.0).

The multilevel analysis showed a lower usage rate of pharmacist consultations for participants in the intervention group than in the control group. The intervention (fixed effect, *P* = .002) and time course (fixed effect, *P* = .004) were significant in the model, but there was no significant interaction term. There were no significant differences between the two groups in the use of other healthcare services provided by family physicians, community health nurses, physiotherapists, and occupational therapists during the 9-month intervention period.

## Discussion

In this longitudinal clinical trial, a positive effect on the incidence of falls, acute events, and hospitalizations demonstrated the benefit of an APN intervention for community-dwelling older people aged 80 and older, although the main hypothesis—that the intervention would have a positive effect on quality of life—was not supported. The missing effect on quality of life is reflective of the heterogeneity of individual regression plots observed during the multilevel analysis of the longitudinal data. Many of the participants had chronic conditions and experience symptoms over years, whereas others had fewer health problems. Individual fluctuations in quality of life were observed during data collection and depended more on symptoms, changed health conditions, and changes in social support than on the APN intervention. This is consistent with the findings of other investigations^48^,^49^ that documented how difficult it is to demonstrate an effect on quality of life in a heterogeneous population at risk of experiencing sudden changes in their health and an overall declining health trajectory. The initial high rating of the participants' quality of life was also unexpected. Self-perceived quality of life in this study was significantly higher than the normal ratings for an equivalent population in Germany.^45^ Given such high baseline ratings, it can be assumed that an additional increase is unlikely with the intervention provided in this study. To improve interpretation of the findings, measures of quality of life or well-being should be combined with measures of functional disability. We agree with the statement^15^ that these standardized measures should capture fluctuations that occur with regard to disability and include proxy information of relatives, friends, or healthcare professionals to avoid self-report bias. Because HCP is a multicomponent intervention, information about environmental and housing conditions, social networks, and social support could be included in a more-sophisticated analysis of the data.

The health situation of older persons, especially those with chronic conditions, is easily destabilized. Nursing interventions for disease and symptom management were proven to have positive outcomes in several populations,^1^,^50^–^52^ although a meta-analysis of preventive home visits^11^ showed conflicting findings and questioned this intervention's effectiveness for people aged 80 and older. In addition, another study^48^ found that home visits did not have any significant effect on health complaints, an outcome measure similar to the outcome of acute events in the present study. In contrast, the present study had a significant effect on reducing acute events and is therefore in agreement with the findings of authors who concluded that specialized nursing education at a higher level such as APN is an important component in providing effective interventions.^53^,^54^ Equipped with this level of education, the intervention nurses in the current study used multidimensional clinical assessments as a starting point for their intervention.^25^ Procedures were goal oriented. Personal continuity established trust and enabled person-centered knowledge that allowed for specific follow-up discussions. This supports the potential effect of targeted interventions for this population.

Falls are common in older adults. At 40% in the year before study entry, the incidence of falls was even higher than the 28% to 34% reported in other studies.^55^,^56^ It is likely that the successful reduction of falls in the current study pertains to the multidimensional approach of the intervention, which was based on available evidence.^10^,^57^ Increasing physical exercise and training were goals of the HCP. Additionally, the APNs discussed environmental factors such as bath mats, handrails, door sills, use of walkers, safe performance of everyday tasks, incontinence management, and medication side effects.

Hospitalization rates were lower in the intervention group, but except for pharmacist consultation, no other changes in the use of healthcare services were observed. It can hence be concluded that the APN intervention did not replace other services (family physicians, therapists, community nurses), but was complementary to them. Switzerland has a policy of mandatory health insurance and a well-established system of community nurses and family physicians who provide basic health care for the population aged 80 and older. Because APN interventions are not an established part of this system, the full potential benefit of the APN interventions described in the study could not be observed. It is likely that APN services integrated into an established system would strengthen the effects observed in this study.

### Limitations and Strengths

Ability to generalize the study findings is limited because of the recruitment procedures that resulted in a convenience sample and the fact that the study design did not allow for blinded data collection. To counterbalance the lack of blinding, all possible measures were taken to separate data collection and intervention, but during telephone interviews, participants talked about the interventions and made blinded data collection impossible.

Self-reported measures were used in this study. Because of the large numbers of healthcare organizations involved, it was not feasible to obtain objective data from patient records. Self-reported measures had several disadvantages. First, the 3-month period of data collection increased the chance that events were not recalled and, thus, not reported. If data were incomplete or information was unclear, study nurses contacted participants by telephone and included next of kin to provide the information needed, but self-reported recalls over the telephone without a daily falls diary are a limitation. Over- or underestimation of events is possible. Second, the lack of objective measures precluded cost-effectiveness analyses. From an integrated economic perspective, it can be assumed that HCP has the potential to pay for itself. Over the 9-month period, the intervention for each participant, including preparation and baseline assessments, four home visits, and subsequent follow-up visits, cost approximately the equivalent of $1,250. Nevertheless, a reduction in healthcare expenditures can be assumed, because one hospital admission in every 10 participants was prevented, along with one acute event in every 4.3 participants and one fall in every 7.1 participants receiving the intervention. Although data on costs for acute hospitalization, acute events, and falls are not available, the expenditures for falls are likely to be high, seeing that, in addition to the 69% with moderate consequences (pain, hematomas, concussions), 8.5% of the participants in the sample who experienced falls suffered severe consequences (fractures, open wounds) and needed additional treatment. Further measures on cost effectiveness should be included in future studies. Given the greater priority of prevention and health promotion within the program, expenses due to nursing home admission could have been avoided. Greater effort through interprofessional collaboration served to pool resources and might have contributed to reduce expenditures. The implementation of HCP on a city-wide level is currently being discussed. Any implementation will include an assessment of the cost effectiveness.

One of the strengths of this study was the use of the principles of health promotion, empowerment, partnership, and family-centeredness to guide the intervention. Intervention nurses were asked to establish an ongoing caring relationship with the participants during the 9-month intervention. This approach explains why withdrawal from the study was low (n = 22, 4.7%), with two-thirds occurring within the first month of the intervention.

Although evidence-based protocols guided the health consultation sessions, they were also customized to the participants' individual situations. Participants' goals were taken into account, activities were negotiated, and their usefulness for and feasibility in everyday life were evaluated during the following sessions.

## Conclusions

In this randomized clinical trial, lower incidence of falls, hospitalization rate, and occurrence of acute events were the positive effects of a 9-month intervention by APNs. The heterogeneous and unstable health situation of the population of persons aged 80 and older, along with the high baseline value, could explain the lack of the hypothesized positive effect on quality of life. Further research is necessary to confirm these findings, using objective outcome measures and different doses of APN interventions within different time frames.
